# 宣威女性肺癌患者肺组织中PAHS-DNA加合物的表达

**DOI:** 10.3779/j.issn.1009-3419.2010.05.25

**Published:** 2010-05-20

**Authors:** 凯云 杨, 云超 黄, 光强 赵, 玉洁 雷, 昆 王

**Affiliations:** 650118 昆明，昆明医学院第三附属医院（云南省肿瘤医院）胸心血管外科 Department of Thoracic Cardiovascular Surgery, Yunnan Tumor Hospital (the Third Affiliated Hospital of Kunming Medical College), Kunming 650118, China

**Keywords:** PAH-DNA加合物, 宣威, 女性肺肿瘤, 免疫荧光, Polycylic aromatic hydrocarbons adduct, Xuanwei, Female lung neoplasms, Immunofluorescence

## Abstract

**背景与目的:**

宣威地区燃煤污染被认为是当地女性肺癌高发的主要原因。本研究旨在通过检测PAH- DNA加合物在宣威女性肺癌患者肺组织中的表达，探讨宣威地区燃煤污染产生的大量多环芳烃类物质与当地女性肺癌的关系。

**方法:**

收集2007年7月-2008年11月云南省肿瘤医院经手术切除的宣威地区女性肺癌患者20例、宣威地区男性肺癌患者20例、非宣威地区女性肺癌患者20例、宣威地区女性肺良性病变患者10例、非宣威地区女性肺良性病变患者10例，肺癌患者取癌组织、癌旁组织、正常肺组织，肺良性病变患者取正常肺组织，总计患者80例，组织200份。采用免疫荧光法检测组织中PAH-DNA加合物的表达，IPP 6.0软件进行图像分析及半定量测定，SPSS 13.0统计软件进行统计学处理。

**结果:**

PAH-DNA加合物在宣威女性肺癌患者癌组织、癌旁组织和正常肺组织中的表达阳性率（90%、80%、65%）高于宣威男性肺癌患者（35%、30%、30%）及非宣威女性肺癌患者（20%、15%、10%）（*P* < 0.01）；在宣威女性肺良性病变患者（阳性率70%）肺组织中的表达也高于非宣威女性肺良性病变患者（阳性率10%）；在癌组织、癌旁组织、正常肺组织中的表达有逐渐降低的趋势，但差别无统计学意义（*P* > 0.05）。

**结论:**

PAH-DNA加合物在宣威女性肺组织中的表达较高，而在宣威男性、非宣威女性中表达较低。

## Abstract (en)

**Background and objective:**

The coal-fired pollution in Xuanwei area has been considered to be local main reason for high incidence of female lung cancer. The aim of this study is to explore the expression of PAH-DNA adducts in lung tissues of Xuanwei female lung cancer patients and to explore the relationship between the large number of coal-fired pollution PAHs materials and the high incidence of Xuanwei female lung cancer.

**Methods:**

We totally collected each 20 cases of Xuanwei female lung cancer patients, Xuanwei male lung cancer patients, Non-Xuanwei female lung cancer patients and collect each 10 cases of Xuanwei, Non-Xuanwei female patients with benign lung lesions. The cancer tissues, adjacent cancer tissues and normal lung tissues were collected in lung cancer patients and only the normal tissues were collected in benign lung lesion patients. There were total 80 cases and 200 tissues. Immunofluorescence was used to detect the expression of PAHDNA adducts in each group. Image pro-plus 6.0 software was used to analyze the images and part quantified analysis. SPSS 13.0 statistical software was used to analyze the data.

**Results:**

The positive expression of PAH-DNA adducts in lung cancer tissues, adjacent cancer tissues and normal lung tissues of Xuanwei female lung cancer patients were 90%, 80% and 65%. They were higher than the positive expression of PAH-DNA adducts in Xuanwei male lung cancer patients (35%, 30%, 30%) and NonXuanwei female lung cancer patients (20%, 15%, 10%)(*P* < 0.01). The expressions in lung tissues of Xuanwei female benign lung lesion patients (positive expression is 70%) were higher than it in Non-Xuanwei female benign lung lesion patients (positive expression is 10%). With the direction changing from cancer tissues, adjacent cancer tissues to normal lung tissues, the expressions of PAH-DNA adducts were decreased but had no statistical difference (*P* > 0.05).

**Conclusion:**

The expressions of PAHDNA adducts in lung tissues of Xuanwei female were higher than which in Xuanwei male and Non-Xuanwei female.

多环芳烃类物质是公认的化学致癌物之一。云南省宣威地区女性肺癌高发，其女性肺癌的平均调整死亡率高达120/10万，宣威市来宾镇女性肺癌死亡率甚至达到400/10万人^[1]^，宣威地区室内燃煤污染被认为是宣威地区女性肺癌高发的主要原因之一，燃煤产生的大量多环芳烃物质可能是当地女性肺癌发病的主要危险因素。研究^[2]^表明宣威地区燃煤家庭室内空气中苯并芘浓度比卫生标准准许浓度（0.1 μg/100m^3^）高很多，最高达到626 μg/100m^3^，超过卫生标准6 000多倍，当地妇女主要从事家务劳动，长期在煤烟污染中生活，受多环芳烃类物质影响尤为严重。我们采用免疫荧光（immunofluorescence）方法检测宣威女性肺癌患者、宣威男性肺癌患者、非宣威女性肺癌患者以及肺良性病变患者肺组织中多环芳烃DNA加合物表达的差异，探讨多环芳烃类物质与宣威女性肺癌的关系。

## 1 材料与方法

### 1.1 临床资料

① 实验组：收集2007年7月-2008年11月云南省肿瘤医院胸心血管外科及胸外科经手术切除的宣威地区女性肺癌患者癌组织、癌旁组织（距癌组织边缘的距离 < 5 cm）和正常肺组织（距癌组织边缘的距离 > 5 cm）各20份。其中鳞癌6例，腺癌14例，年龄为31岁-72岁，平均为52.1岁；②对照组1：同期收集宣威地区男性肺癌患者癌组织、癌旁组织和正常肺组织各20份。其中鳞癌13例，腺癌7例，年龄为35岁-74岁，平均为55.7岁；③对照组2：同期收集非宣威地区女性肺癌患者癌组织、癌旁组织和正常肺组织各20份。其中鳞癌8例，腺癌12例，年龄为36岁-68岁，平均为54.3岁，患者来自昭通、宝山、昆明、丽江以及贵州盘县等地区；④对照组3：同期收集宣威地区女性肺良性病变患者正常肺组织10例。年龄为29岁-61岁，平均为53.7岁；⑤对照组4：同期收集非宣威地区女性肺良性病变患者正常肺组织10例。年龄为32岁-63岁，平均为54.2岁，患者来自昆明、大理、玉溪等地区。

全部病例均进行问卷调查，包括性别、年龄、居住情况、生活习惯、燃煤情况、房屋住宅情况、饮食习惯、家庭经济情况等，并复印患者胸片、B超、CT，以及纤维支气管镜、痰查细胞学检查、术后病检结果。所有病例均不吸烟，也未进行过放疗及化疗。

### 1.2 方法

① 手术取患者癌组织、癌旁组织、正常肺组织放置于-80 ℃冰箱保存；②组织脱水，透明，浸蜡，常规石蜡切片机切片：厚度为3 μm；③切片采用免疫荧光法染色，并进行荧光显微镜摄片及图片分析。（一抗：苯并芘共价加合物单克隆抗体Benzo[a]pyrene(BAP-13): sc-51508；荧光二抗：羊抗鼠IgG-FITC: sc-2010。均购自美国Santa Cruz公司）。

### 1.3 结果判断

PAH-DNA加合物在荧光显微镜下表现为绿色明亮的荧光，主要存在于细胞核，部分存在于细胞质。根据荧光强度分为：①强阳性（+++）：荧光闪亮，呈现明显的亮绿色；②中度阳性（++）：荧光明亮，呈黄绿色；③弱阳性（+）：荧光较弱，但清楚可见；④阴性（－）：无荧光可见。

### 1.4 半定量测定

用IPP 6.0软件进行图像分析。把图像中呈现黄色荧光区域作为AOI（area of interest）进行光密度测定分析。选择测量面积（area）平均光密度（density mean）和累积光密度（integrated optical, IOD），结果以IOD值作为统计数据。

### 1.5 统计方法

应用SPSS 13.0软件包进行卡方检验，*P* < 0.05为差异有统计学意义。

## 2 结果

### 2.1 宣威地区女性肺癌患者与宣威地区男性肺癌患者肺组织中多环芳烃DNA加合物的表达

多环芳烃DNA加合物在细胞中主要表达于细胞核，部分表达与细胞质，其在宣威女性肺癌患者肺组织中的表达较宣威男性肺癌患者高，差异有统计学意义（*P* < 0.05）（表 1）。

**1:** 多环芳烃DNA加合物在宣威女性及男性肺癌患者肺组织中的表达 The expression of PAH-DNA adducts in lung cancer tissues of Xuanwei female and male patients

Patient	*n*	Cancer tissues				Adjacent cancer tissues				Normal tissues		
		+	-	IOD		+	-	IOD		+	-	IOD
Female	20	18	2	6 134.30±32.51		16	4	3 602.37±23.01		13	7	4 482.24±11.07
Male	20	7	13	497.41±8.05		6	14	960.42±11.23		6	14	400.00±5.89
*X*^2^		12.907				10.101				4.912		
*P*		<0.001		<0.001		0.001		<0.001		0.027		<0.001
IOD: integrated optical.												

### 2.2 宣威地区女性肺癌患者与非宣威地区女性肺癌患者肺组织中多环芳烃DNA加合物的表达

多环芳烃DNA加合物在宣威女性肺癌患者肺组织中的表达较非宣威女性肺癌患者高，差异有统计学意义（*P* < 0.05）（表 2）。

**2:** 多环芳烃DNA加合物在宣威女性及非宣威女性肺癌患者肺组织中的表达 The expression of PAH-DNA adducts in lung tissues of Xuanwei female and Non-Xuanwei female patients

Patient	*n*	Cancer tissues				Adjacent cancer tissues				Normal tissues		
		+	-	IOD		+	-	IOD		+	-	IOD
Xuanwei	20	18	2	6 134.30±32.51		16	4	3 602.37±23.01		13	7	4 482.24±11.07
Non-Xuanwei	20	4	16	308.76±6.13		3	17	352.15±4.32		2	18	193.03±5.90
*X*^2^		19.798				16.942				12.907		
*P*		<0.001		<0.001		<0.001		<0.001		<0.001		<0.001

### 2.3 正常肺组织中多环芳烃DNA加合物的表达

多环芳烃DNA加合物在宣威女性正常肺组织中表达较高，在宣威男性、非宣威女性中的表达较低（表 3）。

**3:** 多环芳烃DNA加合物在正常肺组织中的表达 The expression of PAH-DNA adducts in lung normal tissues

	PAH-DNA adducts		*n*	IOD	*P*
	+	-			
Xuanwei female cancer	13	7	20	4 482.24±11.07	—
Xuanwei male cancer	6	14	20	400.00±5.89	0.027
Non-Xuanwei female cancer	2	18	20	193.03±5.90	<0.001
Xuanwei female					
Benign lung lesion	7	3	10	5 738.44±10.35	0.784

### 2.4 多环芳烃DNA加合物在癌组织、癌旁组织、正常组织中的表达趋势

PAH-DNA加合物在癌组织、癌旁组织、正常组织中的表达有逐渐降低的趋势，但差异无统计学意义（表 4，图 1）。

**4:** 多环芳烃DNA加合物在癌组织、癌旁组织、正常组织中的表达趋势 The expression of PAH-DNA adducts in cancer, adjacent and nomal tissues

	PAH-DNA adducts		*n*	IOD	*P*
	+	-			
Cancer	29	31	60	1 808±105.28	0.235
Adjacent cancer	25	35	60	1 726±89.91	
Normal tissues	21	39	60	1 701±81.23	

**1:**
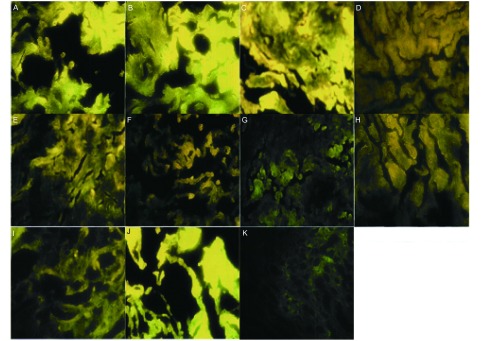
PAH-DNA加合物的表达（×400）。A：宣威女性肺癌癌组织；B：宣威女性肺癌癌旁组织；C：宣威女性肺癌正常肺组织；D：宣威男性肺癌癌组织；E：宣威男性肺癌癌旁组织；F：宣威男性肺癌正常肺组织；G：非宣威女性肺癌癌组织；H：非宣威女性肺癌癌旁组织；I：非宣威女性肺癌正常肺组织；J：宣威女性肺良性病变患者肺组织；K：非宣威女性肺良性病变患者肺组织。 The expression of PAH-DNA adducts (×400). A: Tumor tissue of Xuanwei female lung cancer patient; B: Adjacent tumor tissue of Xuanwei female lung cancer patient; C: Normal tissue of Xuanwei female lung cancer patient; D: Tumor tissue of Xuanwei male lung cancer patient; E: Adjacent tumor tissue of Xuanwei male lung cancer patient; F: Normal tissue of Xuanwei male lung cancer patient; G: Tumor tissue of Non-Xuanwei female lung cancer patient; H: Adjacent Tumor tissue of Non-Xuanwei female lung cancer patient; I: Normal tissue of Non-Xuanwei female lung cancer patient; J: Normal tissue of Xuanwei female patient with benign lung lesion; K: Normal tissue of Non-Xuanwei female patient with benign lung lesion

## 3 讨论

近年来女性肺癌发病率、死亡率有逐年增高趋势，在许多国家其增幅甚至高于男性。中国云南省宣威地区一直是女性肺癌的高发区，其女性肺癌死亡率高达120/10万，远远高于中国女性平均水平（22.9/10万），而宣威来宾镇虎头村女性肺癌死亡率竟达到400/10万人。

肿瘤是DNA损伤后修复失败或修复出错的结果，无论是内源性还是外源性的致癌物，几乎均需要经过这一共同的关键步骤才能起致癌作用。而机体暴露于化学致癌物后所致的DNA损伤，最普遍、最重要的表现形式是DNA加合物。

每燃烧1 kg煤产生多环芳烃类物质可达6×10^5^/μg-14 ×10^5^/μg^[3]^。多环芳烃进入机体后经细胞色素P450和环氧化物水解酶、单加氧酶等代谢过程产生反式二羟环氧苯并芘（anti_benzo(a)pyrenediol_epoxide, BPDE），BPDE是直接的强致癌物，可以和DNA共价结合形成多环芳烃_DNA加合物（polycylic aromatic hydrocarbons adduct, PAHDNA adduct），或与蛋白质结合形成多环芳烃蛋白加合物。当基因复制到多环芳烃_DNA加合物位点时，则不能继续复制，还可能发生突变或碱基缺失。多环芳烃蛋白加合物则通过反式作用因子来影响DNA复制及转录。当关键基因突变导致细胞失去正常调控时，就会引起细胞癌变^[4]^。多环芳烃与其它毒物一样，在体内要经过代谢活化，产生生物活性，并经过代谢失活，排出体外。因此，代谢酶活性的强弱对多环芳烃的生物效应影响很大。PAH-DNA加合物不仅能反映出人体总的多环芳烃接触量，还能反映出多环芳烃经人体代谢后致突变作用的实际含量，因此PAH-DNA加合物既是多环芳烃的接触指标，又是其致癌作用的效应指标^[5]^。

多环芳烃类物质与肺癌的关系国内外都已经研究得很多，但云南宣威地区却有其特殊性。宣威地区女性几乎不吸烟，但其肺癌发病率却非常高，某些地区甚至超过男性。现认为主要与当地的室内燃煤污染有关。

宣威市位于滇东北部乌蒙山区，是云南省主要产煤基地，小煤窑遍及全县，农民世世代代在室内挖坑为炉，多数家庭燃烧烟煤取暖做饭，炉子温度不高，不能完全燃烧煤炭，加之室内通风不良，当地女性长年累月在烟雾中生活，受燃煤污染影响特别严重。相对室外来说，室内通风不畅可导致燃煤空气污染比室外高出几十甚至上百倍。WHO公布，柏林市中心空气中可吸入颗粒物（particles with diameters of 10 μm or less, PM10）浓度为30 μg/m^3^，Bangkok路边空气中PM10浓度为240 μg/m^3^，而直接用火塘燃煤的室内空气中PM10浓度高达3 000 μg/m^3^^[6]^。云南省疾病预防控制中心监测的结果发现：70%的时间段，宣威地区户外空气质量水平达国家标准^[7]^。然而燃烟煤农户室内空气中BaP浓度高达626 μg/100m^3^，超过其建议卫生标准6 000多倍，同时也远远超过焦炉顶工人暴露水平^[8]^。

蓝青、何兴舟等^[9]^研究发现：云南宣威地区煤层属于晚二叠纪煤层，煤炭品种分类为烟煤。宣威地区烟煤燃烧产生的空气悬浮颗粒物较无烟煤多，并且这些空气悬浮颗粒物中有机物含量（占43%）也远远高于无烟煤，这些有机物大部分为多环芳烃类物质^[10]^。宣威肺癌死亡率分布也呈现明显的地区差异，肺癌死亡率高低与使用燃料品种有关。烧烟煤死亡率最高，木柴、无烟煤次之。肺癌死亡率性别比值（男:女）各地差异较大，荷兰为13.5:1，美国为1.8:1，中国为2.07:1，云南宣威地区为1.09:1，宣威来宾镇为0.87:1。烧烟煤人群肺癌死亡年龄高峰提前，烧烟煤人群肺癌死亡率是非烧烟煤人群的1.70倍^[11]^。

宣威地区流行病学研究^[12]^发现：烧烟煤人群患肺癌危险性是非烧烟煤人群的6.05倍（OR=6.05）。回顾性队列研究^[13]^发现：女性中烧烟煤人群肺癌死亡率是非烧烟煤人群肺癌死亡率的25.6倍（RR=25.6），归因百分比为96%。暴露剂量反应关系研究^[14]^发现：伴随1958年前烧烟煤人口百分比升高（0%-100%）肺癌年龄调整死亡率相应升高（2.08/10^5^-174.21/10^5^）；伴随室内空气中BaP浓度升高（356.0 ng/m^3^-2 485 ng/m^3^）肺癌死亡率相应升高（2.08/10^5^-174.21/10^5^）。

研究发现：多环芳烃代谢酶多态性对宣威地区女性影响也较男性大。GSTM1在日本、欧洲和北美人群中表达的比例高达40%-50%，在中国为35%-63%，在云南省宣威市女性人群中为34.2%，其相对危险度为2.39（1.25-4.56），高于男性^[15]^。

室内燃煤空气中以苯并（a）芘为代表的大量致癌性多环芳烃类物质可能是宣威女性肺癌发病的主要危险因素之一，多环芳烃代谢酶的基因多态性可能是宣威地区女性肺癌发生发展的遗传因素，这与我们研究的结果相符合。现在，宣威地区女性肺癌发病仍然呈增高趋势，而且还呈年轻化趋势，我们收治的宣威肺癌患者中，20多岁的人群屡见不鲜。我们认为，通过研究宣威地区女性肺组织中多环芳烃DNA加合物的表达，可以帮助我们进一步了解当地肺癌高发与燃煤污染、多环芳烃类物质污染的关系，并指导制定进一步的预防措施。
